# Supplementary material for: Modeling the systemic risks of COVID-19 on the wildland firefighting workforce
Source: Sci Rep. 2022 May 18;12:8320. doi: 10.1038/s41598-022-12253-x (PMC9116702; doi:10.1038/s41598-022-12253-x)
Supplement: Supplementary file 1 — Supplementary Information. [file 41598_2022_12253_MOESM1_ESM.docx]

**Technical Appendix**

Here in the technical appendix we include a detailed outline of the agent-based algorithm, a list of the specific parameters we used within the algorithm with sources for the parameters cited (where applicable), a description of how we processed the assignment data and a summary of the 2016-2018 fire seasons including comparisons between model runs, sample size comparisons, and a description of our calibration of the reproductive parameters we used for SARS-CoV-2.

*Agent-based model algorithm*

1. Assign agents to their current incident and module.
   1. Agents not on an assignment are assigned to off-fire status.
   2. Leadership status is assigned for agents in modules on new assignments.
2. Simulate contacts between agents in each module, including the management modules. For each module:
   1. Identify all agents who are not isolated and are infectious (symptomatic or asymptomatic).
   2. Identify all agents who are not isolated and are susceptible or exposed.
   3. Given at least one infectious, not isolated agent in the module and at least one susceptible agent, calculate average number of infectious contacts (*a_m,d_*) that occur for an individual in module *m* on day *d* using the following equation, where $\beta_{sym}$ is the daily transmission number for symptomatic agents, $\beta_{asym}$ is the daily transmission number for asymptomatic agents, *c* is the contact intensity multiplier for modules, and $n_{s,m,d}$ is the number of agents in the module *m* in state *s* on day *d*.
      $a_{m,d}=\frac{c*\left( \left( \beta_{sym}*n_{sym,m,d} \right)+ \left( \beta_{asym}*n_{asym,m,d} \right) \right)}{n_{not isolated,m,d}}$
   4. For each susceptible agent, simulate the number of infectious contacts from other module members today using a draw from a Poisson distribution with the mean of the distribution (lambda) equal to $a_{m,d}$. The viral state is changed from susceptible to exposed for each agent whose random draw is greater than zero.
3. Model contacts between module leaders
   1. Identify all module leaders who are not isolated and are infectious (symptomatic or asymptomatic)
   2. Identify all module leaders who are not isolated and are susceptible or exposed
   3. Given at least one infectious, not isolated leader and at least one susceptible leader, calculate calculate average number of infectious contacts (*a_l,d_*) that occur for an individual designated as a leader (*l*) on day *d* using the following equation where $\beta_{sym}$ is the daily transmission number for symptomatic agents, $\beta_{asym}$ is the daily transmission number for asymptomatic agents, and $n_{s,l,d}$ is the number of agents designated as leaders in state *s* on day *d*.
      $a_{l,d}=\frac{\left( \left( \beta_{sym}*n_{sym,l,d} \right)+ \left( \beta_{asym}*n_{asym,l,d} \right) \right)}{n_{not isolated,l,d}}$
   4. For each susceptible leader, simulate the number of infectious contacts from other leaders today using a draw from a Poisson distribution with the mean of the distribution (lambda) equal to $a_{l,d}$. The viral state is changed from susceptible to exposed for each leader whose random draw is greater than zero.
4. Model infectious contacts for off-fire agents.
   1. Identify all off-fire agents.
   2. For each agent, draw a random number from a Uniform(0,1) distribution.
   3. Compare the random draws to the off-fire infection parameter (*eir*). If the random draw is less than the off-fire infection parameter and the agent is susceptible, then the agent’s viral state changes to exposed.
5. Some exposed agents become infectious.
   1. Identify all agents in the exposed viral state.
   2. For each agent, draw two random numbers from a Uniform(0,1) distribution. The first draw will be used to determine if the agent is leaving the exposed state. The second draw will be used to determine which infectious state (symptomatic or asymptomatic) the agent enters, if they do leave the exposed state.
   3. Compare the random draw values to the daily probability of leaving the exposed state (*p_e_*=1/*D_e_*), where *D_e_* is the average incubation period, and the probability of being symptomatic (*p_i_*). There are three possible outcomes for the agent:
      1. If the first random draw is less than the daily probability of leaving the exposed state and the second random draw is less than the probability of being symptomatic, then the agent’s viral state changes to symptomatic.
      2. If the first random draw is less than the daily probability of leaving the exposed state and the second random draw is greater than or equal to the probability of being symptomatic, then the agent’s viral state changes to asymptomatic.
      3. If the first random draw is greater than or equal to the daily probability of leaving the exposed state, then the agent’s viral state does not change.
6. Some infectious agents recover
   1. Identify all agents in the symptomatic or asymptomatic viral state.
   2. For each agent, draw a random number from a Uniform(0,1) distribution.
   3. Compare the random draw to the daily probability of recovering (*p_r_*). If the random draw is less than the daily probability of recovery, then the agent’s viral state changes to recovered.
7. Some infectious agents isolate.
   1. Identify all symptomatic and asymptomatic agents who are not currently isolated.
   2. For each agent, draw a random number from a Uniform(0,1) distribution.
   3. Compare the random draw value to the daily probability that an agent correctly identifies their symptoms and/or receives a positive test (*p_IQ_* for symptomatic agents and *p_AQ_* for asymptomatic agents). If the random draw is less than the daily probability that an agent correctly identifies their symptoms and/or receives a positive test then the agent’s isolation state changes to isolated.
8. Isolate other agents, increment isolation day counts, and release agents from isolation.
   1. Identify all isolated agents.
   2. If the agent is on an assignment and part of a crew module, then isolate all other non-isolated module members.
   3. For all isolated agents, increment the number of days they have been isolated by one.
   4. If an agent has been isolated for greater than the number of required isolation days (*D_q_*) and their viral state is not symptomatic then their isolation state changes to not isolated and their isolation day count is set to 0. If the agent is still symptomatic after the required number of isolation days, then they continue to be isolated until they move to the recovered state (driven by *p_r_*, the daily probability of recovery).
9. Vaccinate agents.
   1. Identify all agents that are not isolated and not vaccinated.
   2. Randomly sample these agents to determine who is vaccinated on that day, vaccinating exactly the number of agents specified for that geographic area on that day (*vo_g,d_* for management and *vno_g,d_* for crew).
   3. Sample the newly vaccinated agents whose viral state is susceptible to determine if their viral state changes from susceptible to recovered, changing the states for exactly the number specified by the vaccination efficacy parameter (*ve* * *vo_g,d_* and *ve* * *vno_g,d_*).
10. If this is not the last day of the season, increment forward one day and go back to step 1. If this is the final day of the season, save the following information for each resource on each day of the season: viral state, vaccination state, isolation state, and leader status.

*Parameters used in the agent-based model*

The parameters used in the simulations are listed in Tables 1 and 2. Where appropriate, we also list references for parameter values.

| Parameter and description | Baseline | Low Compliance | High Compliance | Parameter use |
| --- | --- | --- | --- | --- |
| *eir*: The probability a firefighter who is off-assignment will be infected by COVID-19 | 0.00042 | 0.00084 | 0.00021 | Compared to a Uniform(0,1) random draw: if the draw is <= *eir* the agent is assigned an infectious viral state |
| $\beta_{sym}$ | 0.13 | 0.177 | 0.077 | Used to calculate the average number of infectious contacts that lead to infection among a non-infectious agent incurs for a single day |
| *V_management,init_*: Initial number of vaccinated individuals in the management population | 50% | 50% | 70% | Used to calculate the number of management agents who are assigned vaccinated status on the first day of the season. Agents are sampled randomly. |
| *V_management,final_*: final number of vaccinated individuals in the management firefighting population | 75% | 50% | 90% | Used to create a linear function that calculates the number of management agents who are vaccinated daily for each GACC (*vo_g,d_*) between the start of the season and the final vaccination date. Agents are sampled randomly. |
| *V_crew,init_*: Initial number of vaccinated individuals in the crew firefighting population | 50% | 50% | 50% | Used to calculate the number of crew agents who are assigned vaccinated status on the first day of the season. Agents are sampled randomly. |
| *V_crew,final_*: final number of vaccinated individuals in the crew firefighting population | 50% | 50% | 75% | Used to create a linear function that calculates the number of crew agents who are vaccinated daily (*vno_g,d_*) between the start of the season and the final vaccination date. Agents are sampled randomly. |
| Final vaccination date | July 1 | First day of the season | July 1 | Used to create the linear function determining how many agents are vaccinated daily to reach the final vaccination population as specified by *V_ crew,final* and *V_ management,final* |
| *p_IQ_*: Probability that a symptomatic agent who is not quarantined recognizes symptoms and moves to quarantine | 0.5 | 0.3 | 0.7 | Compared to a Uniform(0,1) random draw: if the draw is <= *p_IQ_* then the agent’s isolation state is changed to isolated |

Table 1: Parameter values that vary across scenarios

| Parameter | Value | Description | Parameter use |
| --- | --- | --- | --- |
| *p_i_* | 0.4286 | Probability that an infectious agent is symptomatic (weighted by population age)[1] | Compared to a Uniform(0,1) random draw: if the draw is <= *p_i_* then the agent’s viral state is changed to symptomatic, else the viral state is changed to asymptomatic |
| *p_AQ_* | 0 | Proportion asymptomatic that quarantines due to a positive test | Compared to a Uniform(0,1) random draw: if the draw is < *p_AQ_* then the agent’s isolation state is changed to isolated |
| *R_init_* | 25% | Initial percent of recovered individuals in the firefighting population | Used to calculate the number of agents who start the season in the recovered viral state. Agents are sampled randomly. |
| *Num leaders* | 4 | Number of leaders per module (crew) | Calculates the number of agents who are assigned leadership status for crew modules. |
| *I_init_* | 0.598% | Initial percent of infectious individuals in the firefighting population | Used to calculate the number of agents who start the season in the symptomatic viral state. Agents are sampled randomly. |
| *D_e_* | 5 | Average incubation period[2], [3] | Used to calculate *p_e_*, the probability of leaving the exposed state, which is compared to a Uniform(0,1) random draw: if the draw is <= *p_e_* then the agent’s viral state will change to either symptomatic or asymptomatic |
| *D_r_* | 8 | Average days from onset of infectiousness to recovery[4], [5] | Used to calculate *p_r_*, the probability of leaving an infectious state, which is compared to a Uniform(0,1) random draw: if the draw is <= *p_r_* then the agent’s viral state will change to recovered |
| *l* | 0.5 | Proportion management designated leaders | Calculates the number of agents who are assigned leadership status for management modules. |
| *c* | 4 | Reproduction multiplier for modules | Used to calculate the average number of infectious contacts that a non-infectious agent incurs for a single day ($a_{m,d}$) |
| *D_q_* | 10 | Required days of isolation[6] | Compared to days an agent has been in isolation: given days in isolation is >= *D_q_* and agent is recovered, agent leaves isolation state. |
| *ve* | 0.95 | Vaccine efficacy[7], [8] | Used to calculate the number of agents who move from symptomatic to recovered upon receiving their vaccine. |

Table 2: Parameter values that stay constant across scenarios

*Assignment data and fire season summaries*

To build our fire assignment dataset, we identified the set of wildland fires that were managed by a Type 1, Type 2, Type 3, National, or Area Command incident management team or incident commander which burned in the US in 2016, 2017, and 2018 using data archived in the Resource Ordering and Status System (see [9]–[11] for previous peer reviewed studies using this data). For each of these fires we obtained the assignments for each of the individuals that provided wildland firefighting capacity or wildland fire management capacity on the fire. Individuals are assigned unique identifiers that are constant across the season, allowing us to identify which days each individual was assigned to each of the large fires and to observe movement of individuals between large fires across the fire season. The home geographic area for each individual is typically provided; if the home geographic area is unknown then personnel are assigned to the geographic area in which they are first assigned to an incident. Individuals are also classified by their role on the fire, that is, they are assigned to a specific hand crew, a crew managing a piece of equipment (for example, engine or dozer), or management (personnel handling the logistics and planning for the fire). We use these roles to create the modules on each fire. We did not include personnel assigned to aerial resources as aircraft assignments in ROSS are not always reliable [10] and those personnel have a lower level of contact with other firefighting personnel.

The 2016 fire season was a fairly average season, with slightly fewer fires and slightly fewer acres burned than the ten-year average, though the number of structures burned was slightly over the yearly average (calculated since 1999). There was a pulse of fire activity early in the season (pre-July) driven by the SW and Southern California, followed by a pause (early July), followed by a medium level of fire activity throughout the rest of the season. The 2016 fire season was unique in having a pulse of destructive fire activity in the Southern Area in December, which accounted for over 2000 of the 4312 structures burned in 2016 [12]. The 2017 fire season was a more severe fire season than 2016, with the number of acres burned well above the ten-year average as well as setting the record for most structures burned (1999-2017). The Northern Rockies, Great Basin, and Northwest all saw substantial fire activity mid-season (August-September). Southern California experienced a very destructive pulse of fire in December [13]. The 2018 fire season was also an above average fire season, with the number of acres burned well above the ten-year average and again setting a record for the number of structures burned (1999-2018). The Northwest, Great Basin, and California experienced substantial fire activity mid-season (July-August) and California again experienced a late season pulse of fire in November [14]. The number of personnel on assignment daily from each Geographic Area is shown in Figure S.1. The assignment data include 190, 233, and 234 large fires in 2016, 2017 and 2018, respectively, with 37,299, 43,360, and 40,593 personnel assigned to at least one of these fires in 2016, 2017, and 2018, respectively.


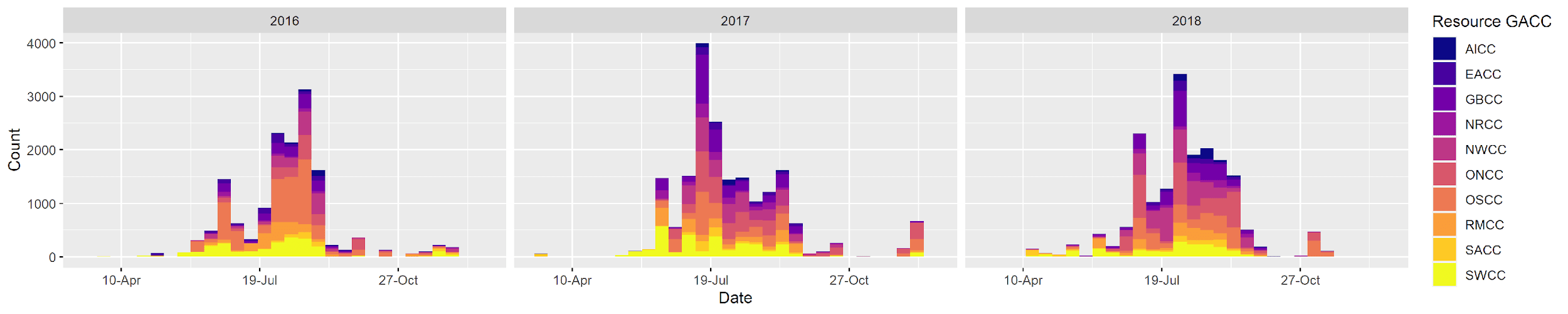


Figure S.1: Number of potential disease spreading reassignments across the 2016, 2017, and 2018 fire season. The color indicates the geographic area from which the firefighter originates. Abbreviations for geographic areas (GACCs) are: AICC (Alaska), EACC (Eastern Area), GBCC (Great Basin), NRCC (Northern Rockies), NWCC (Northwest), ONCC (Northern California), OSCC (Southern California), RMCC (Rocky Mountain Area), SACC (Southern Area), SWCC (Southwest).

Simulations using the Baseline scenario parameters show similar distributions of both infection prevalence over time, cumulative infections (Figure S.1a), and worker days missed (Figure S.1b). The median number of cumulative infections for runs using the 2016, 2017, and 2018 assignments under the Baseline scenario assumptions was 79.5 [IQR: 72-88], 94 [IQR: 81-102], and 94 [IQR: 82.75-108.25] respectively. We do observe a higher level of cumulative infections overall in 2017 and 2018 than 2016; this is because the total number of personnel assigned to a large fire is highest in 2017, leading to a larger pool of personnel that can be infected off fire. The median number of cumulative infections in 2016 was 1498 [IQR: 1471-1521], in 2017 was 1915 [IQR: 1892-1944], and in 2018 was 1808 [IQR: 1782-1849]. Similar to the number of on-fire infections, the number of worker days missed (both when quarantining all personnel and when quarantining only vaccinated individuals) does not vary substantially between years; worker days missed (for quarantining only unvaccinated individuals) was 995.1 [IQR: 801-1244.5], 1007 [IQR: 842-1198] and 1003.5 [IQR: 810.5-1193.5] for 2016, 2017 and 2018, respectively.


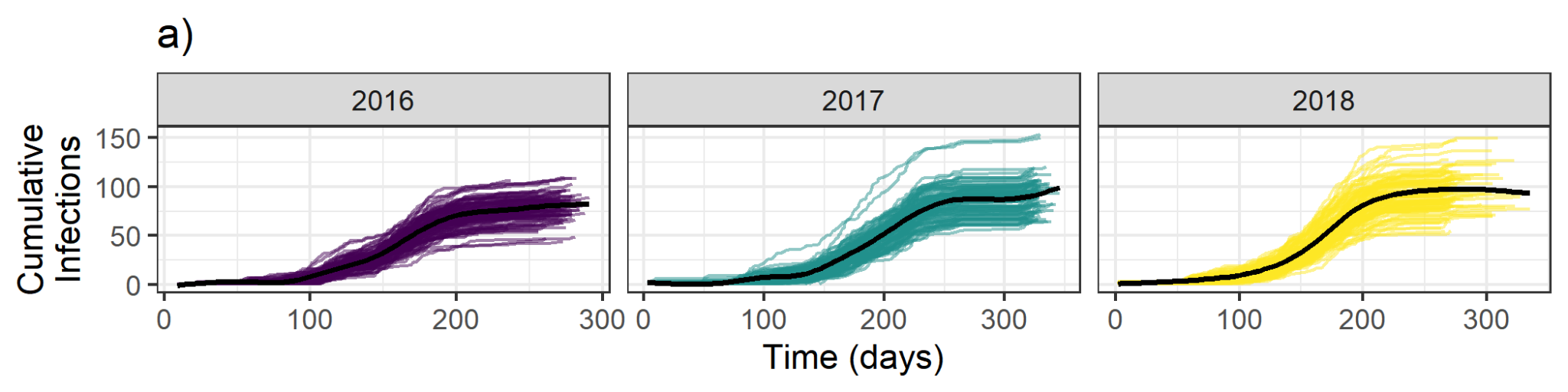


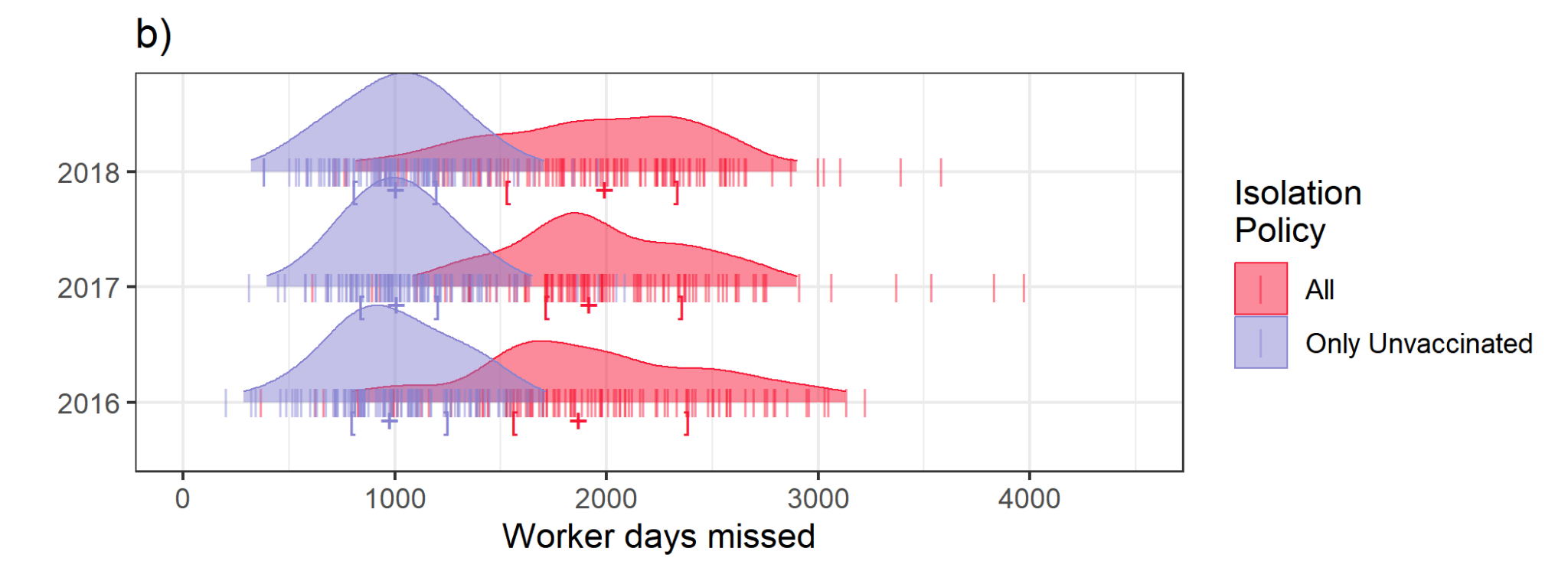


Figure S.2: Prevalence paths for cases of SARS-CoV-2 incurred during a fire assignment (a) and the distribution of worker days missed for (b) using the Baseline scenario parameters occurring for runs using 2016, 2017 and 2018 assignment data.

*Calibration of reproductive parameters for SARS-CoV-2*

A key calculation in the model is the average number of infectious individuals that a single individual contacts at a close enough level to transmit the virus. This can be calculated using the equation from step 1c (reproduced below for ease of reading), where $a_{m,d}$ is the average number of infectious contacts that occur for an individual in module *m* on day *d*, $n_{sym,m,d}$ and $n_{asym,m,d}$ are the number of symptomatic personnel and number of asymptomatic personnel, respectively, in module *m* on day *d* who are not isolated and $n_{not isolated,m,d}$ is the total number of not isolated personnel in module *m* on day *d*. This includes a “reproduction multiplier” (*c*) that allows the intensity of contact to vary depending upon whether the contact is between module members or leaders: contact is substantially higher within modules.

$a_{m,d}=\frac{c \left( \left( \beta_{sym}*n_{sym,m,d} \right)+ \left( \beta_{asym}*n_{asym,m,d} \right) \right)}{n_{not isolated,m,d}}$

Based on estimates of disease spread from those who are asymptomatic relative to those who are symptomatic, we assumed $\beta_{asym}=\frac{2}{3}\beta_{sym}$. To calibrate $\beta_{sym}$ and $\beta_{asym}$we ran the model on the five days with the maximum number of personnel from three fires from 2017 that have previously been used to examine COVID-19 spread through wildland fire personnel: the Lolo Peak fire, the Highline fire, and the Tank Hollow fire. For the Low Compliance scenario, we aimed to provide a reproductive capacity such that each single infectious individuals would infect 1.8 other individuals on average. For the High Compliance scenario, we aimed to provide a reproductive capacity such that each single infectious individual would infect 0.8 other individuals on average. For the Baseline scenario, we aimed to provide a reproductive capacity such that each single infectious individual would infect 1.34 other individuals on average. The final values of $\beta_{sym}$ that we used are listed in Table 2.

*Sensitivity analysis*

Our agent-based model relies on many parameters, some of which are adapted from specific studies and others are based on expert judgment. For example, we spoke to several wildland firefighters about their level of interaction with other firefighters and asked how these interactions may change under the “module as one” model. Because of the model complexity, we designed scenarios that combine changes of several parameters. While the results of the simplified scenarios are easier to understand, the role of specific parameters is not clear in some cases.

We conduct sensitivity analysis of our results to parameters that are uncertain or specific to the wildfire fighting without estimation in the scientific literature. We focus on the rate of off-fire transmission, the rate of self-isolation for firefighters on the fire, and the multiplier that determines the increased rate of transmission for firefighters within the same crew relative to non-crew interactions. Figures S.3, S.4, and S5 illustrate the sensitivity of modeled cumulative infections over the season with respect to the rate of off-fire transmission, the rate of self-isolation for firefighters on the fire, and the multiplier that determines the increased rate of transmission for firefighters within the same crew relative to non-crew interactions.

**Off-fire transmission.** Wildland firefighters return home when not assigned to an incident. Once off duty, firefighters are assumed to behave like any other people in their community and thus face a risk of infection similar to the prevalence of COVID-19 in their community. Our baseline scenario assumes a prevalence of 42 daily cases per 100k, which was approximately the U.S. average in April 2021 (when the paper was drafted). To investigate the model sensitivity, we consider a higher risk associated with higher prevalence of 84 daily cases per 100k (low compliance) and 21 daily cases per 100k (high compliance). These scenarios may also be interpreted as alternative behavioral assumptions where the low compliance case corresponds to firefighters undertaking less precaution than the average person in their community.

Figure S.3 shows that the results are sensitive to the off-fire transmission rate. Indeed, the low compliance scenario results in off-fire cumulative infections exceeding 3000 compared to the baseline of around 2000. Similarly, the high compliance scenario results in nearly 1000 off-fire cumulative infections. Note that variation in this off-fire infection risk also has significant impact on transmission on the fire as some of those infected firefighters arrive at incidents exposed, but not yet infectious. This result is conceptually similar to the role of community transmission of COVID-19 and case rates in K-12 schools with transmission mitigation measures[15]. We conclude that the model results are sensitive to the rate of off-fire transmission.

**Self isolation.** Figure S.4 illustrates that the model is not sensitive to assumptions about self-isolation of symptomatic infectious individuals.

**Module transmission risk.** We took care in modeling “module as one”, one of the key non-pharmaceutical interventions implemented in wildland firefighting operations in 2020. The intent of the policy was to minimize the risk of transmission between modules or crews. The idea was similar to so called pods in other institutionalized settings.

Figure S.5 illustrates that the on-fire transmission is sensitive to the assumed increased transmission risk within the module. Indeed, cumulative on-fire infection was greater under the low compliance case where intra-module spread was assumed 6 times greater than between overhead personnel. However, the difference between the high and low compliance scenarios are relatively small compared to the differences across scenarios presented in the main text.

*
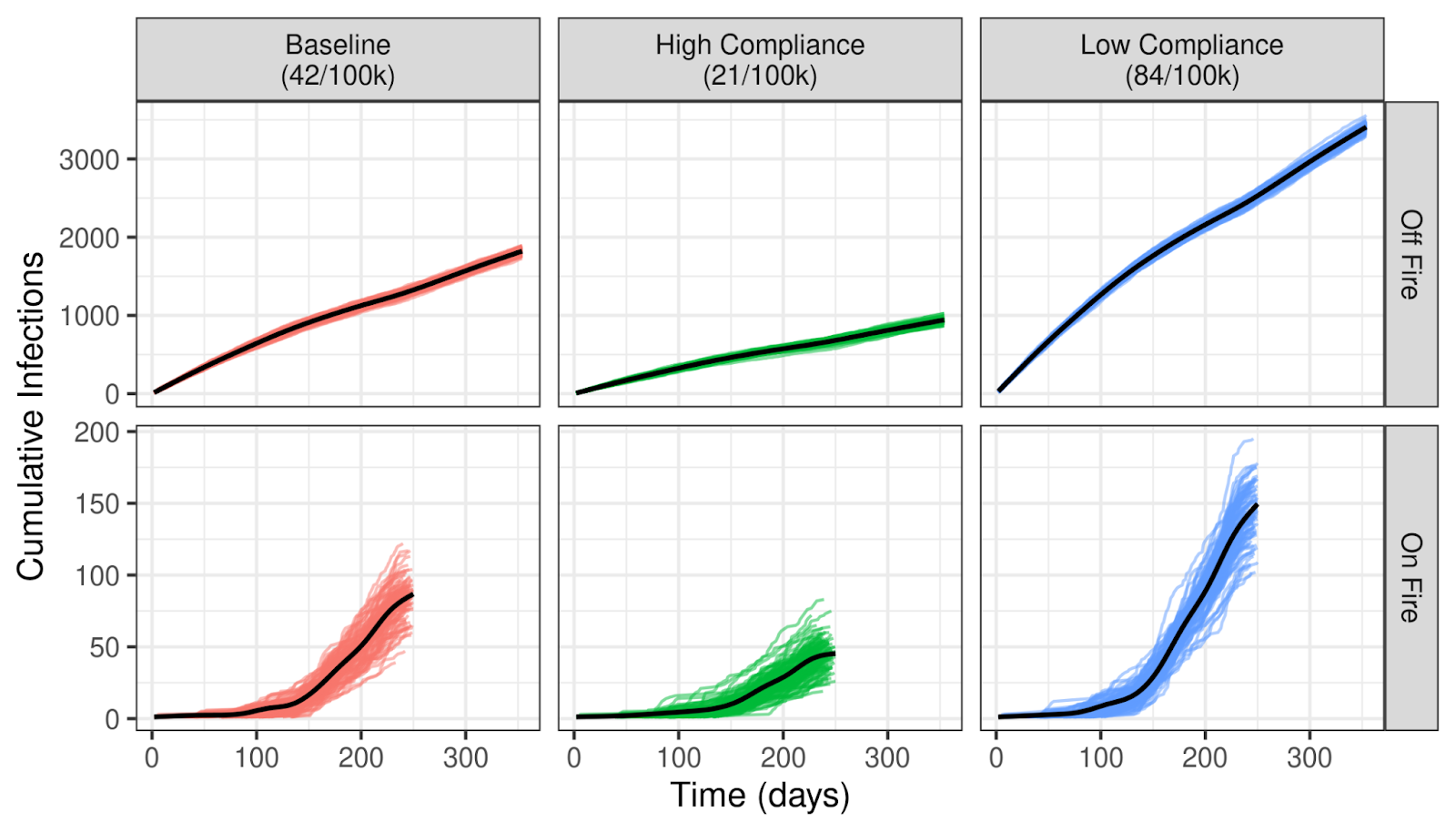
*

Figure S.3  Modeled cumulative infections on and off fire under different levels of infection risk when firefighters are not on duty.  We calibrate infection risk to three levels of prevalence: baseline (42 daily cases /100k), high compliance (21 daily cases / 100k), and low compliance (84 daily cases /100k).


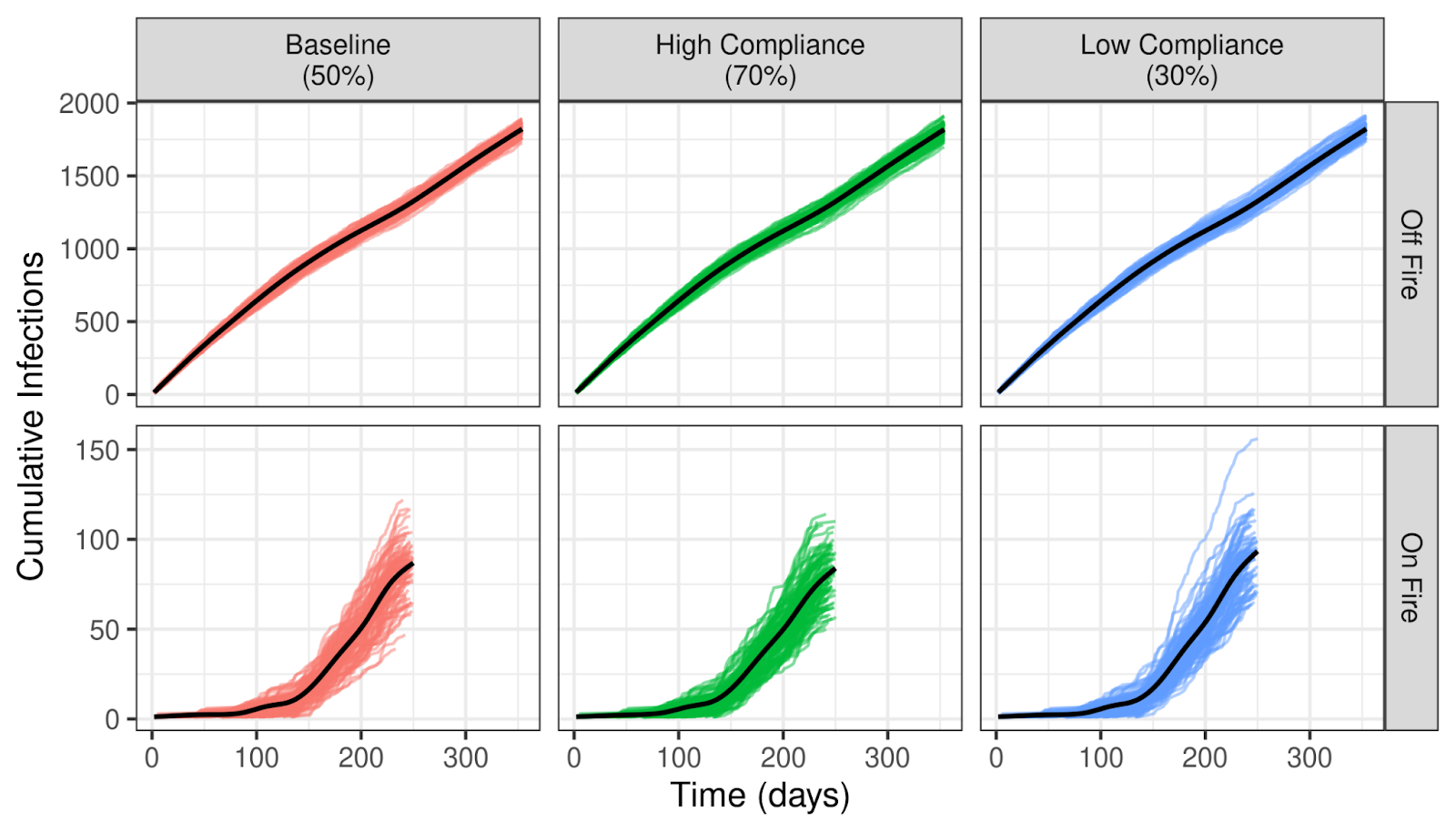


Figure S.4  Modeled cumulative infections on and off fire under different rates of self-isolation: baseline (50%), high compliance (70%), and low compliance (30%).


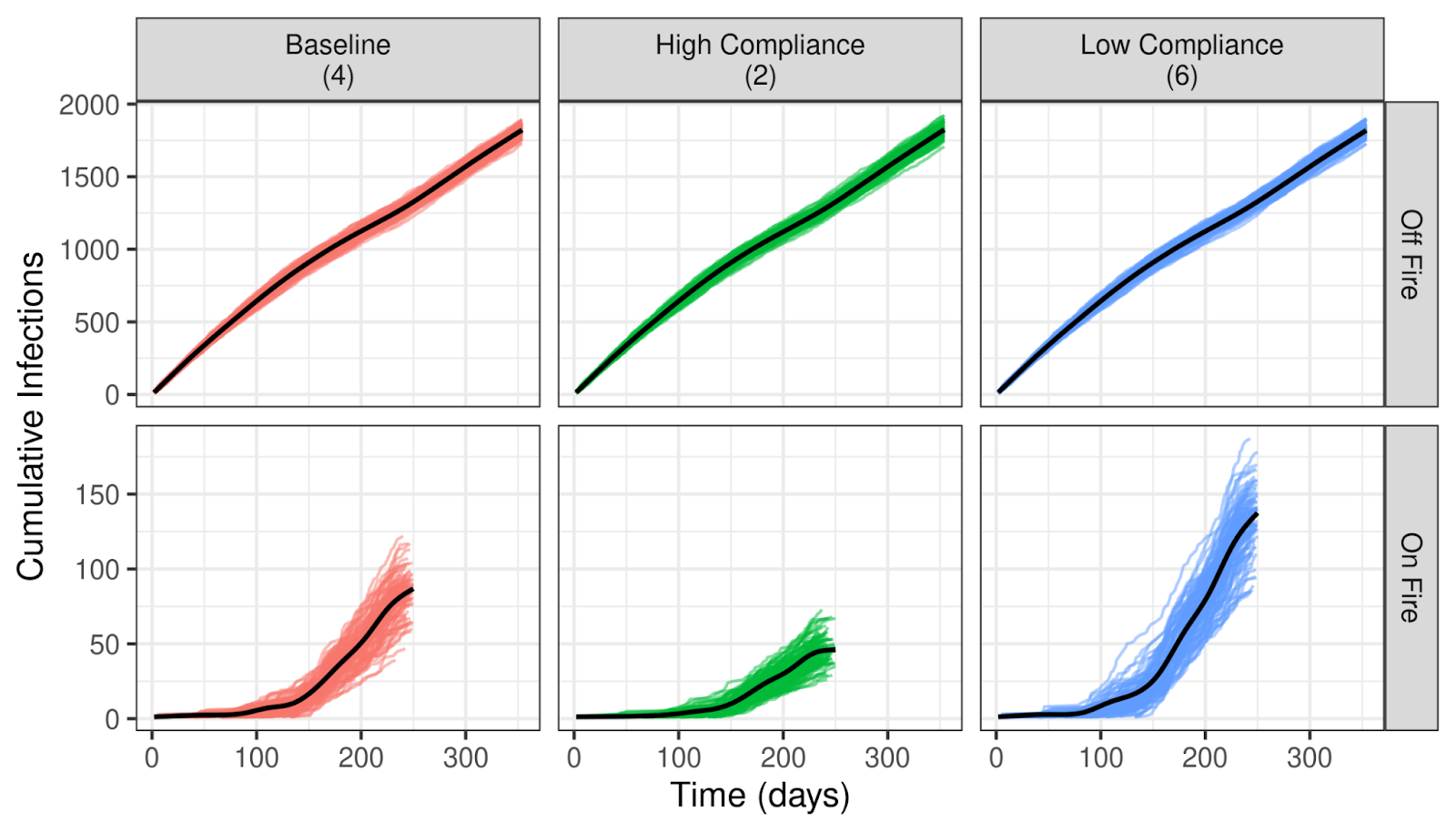


Figure S.5  Modeled cumulative infections on and off fire under different rates of transmission within a crew module: baseline (4x), high compliance (2x), and low compliance (6x).

*Sample size for Monte Carlo simulations*

We wanted to run enough simulations to adequately represent the stochasticity within this system, but to also use computing resources efficiently. We ran sample sets of simulations using both 100 runs and 500 runs. We found the distributions of infections did not vary substantially between 100 and 500 runs (see Table S.3 for comparisons).

| **Scenario** | **Cumulative infections 500 runs** | **Cumulative infections 100 runs** |
| --- | --- | --- |
| Low Compliance | 4506 [IQR: 4454-4556] | 4512 [IQR: 4461-4566] |
| Baseline | 1916 [IQR: 1885-1950] | 1915 [IQR: 1892-1944] |
| High Compliance | 635 [IQR: 619-653] | 634.5 [IQR: 618.8-654.2] |

Table S.3: A comparison of the distribution of cumulative infections from a set of 500 simulation runs and a set of 100 simulation runs using the 2017 assignment data for all three behavioral scenarios.

**References**

[1] N. G. Davies *et al.*, “Age-dependent effects in the transmission and control of COVID-19 epidemics,” *Nat Med*, vol. 26, no. 8, pp. 1205–1211, Aug. 2020, doi: 10.1038/s41591-020-0962-9.

[2] S. A. Lauer *et al.*, “The Incubation Period of Coronavirus Disease 2019 (COVID-19) From Publicly Reported Confirmed Cases: Estimation and Application,” *Annals of Internal Medicine*, vol. 172, no. 9, pp. 577–582, May 2020, doi: 10.7326/M20-0504.

[3] Q. Bi *et al.*, “Epidemiology and transmission of COVID-19 in 391 cases and 1286 of their close contacts in Shenzhen, China: a retrospective cohort study,” *The Lancet Infectious Diseases*, vol. 20, no. 8, pp. 911–919, Aug. 2020, doi: 10.1016/S1473-3099(20)30287-5.

[4] X. He *et al.*, “Temporal dynamics in viral shedding and transmissibility of COVID-19,” *Nat Med*, vol. 26, no. 5, pp. 672–675, May 2020, doi: 10.1038/s41591-020-0869-5.

[5] H.-Y. Cheng *et al.*, “Contact Tracing Assessment of COVID-19 Transmission Dynamics in Taiwan and Risk at Different Exposure Periods Before and After Symptom Onset,” *JAMA Intern Med*, vol. 180, no. 9, p. 1156, Sep. 2020, doi: 10.1001/jamainternmed.2020.2020.

[6] Centers for Disease Control and Prevention, “When You Can be Around Others After You Had or Likely Had COVID-19,” Mar. 12, 2021. https://www.cdc.gov/coronavirus/2019-ncov/if-you-are-sick/end-home-isolation.html (accessed Jun. 26, 2021).

[7] L. R. Baden *et al.*, “Efficacy and Safety of the mRNA-1273 SARS-CoV-2 Vaccine,” *N Engl J Med*, vol. 384, no. 5, pp. 403–416, Feb. 2021, doi: 10.1056/NEJMoa2035389.

[8] F. P. Polack *et al.*, “Safety and Efficacy of the BNT162b2 mRNA Covid-19 Vaccine,” *N Engl J Med*, vol. 383, no. 27, pp. 2603–2615, Dec. 2020, doi: 10.1056/NEJMoa2034577.

[9] M. P. Thompson, J. Bayham, and E. Belval, “Potential COVID-19 Outbreak in Fire Camp: Modeling Scenarios and Interventions,” *Fire*, vol. 3, no. 3, p. 38, Aug. 2020, doi: 10.3390/fire3030038.

[10] E. J. Belval, C. S. Stonesifer, and D. E. Calkin, “Fire Suppression Resource Scarcity: Current Metrics and Future Performance Indicators,” *Forests*, vol. 11, no. 2, p. 217, Feb. 2020, doi: 10.3390/f11020217.

[11] K. M. Lyon, H. R. Huber-Stearns, C. Moseley, C. Bone, and N. A. Mosurinjohn, “Sharing contracted resources for fire suppression: engine dispatch in the Northwestern United States,” *Int. J. Wildland Fire*, vol. 26, no. 2, p. 113, 2017, doi: 10.1071/WF16100.

[12] National Interagency Coordination Center, “National Interagency Coordination Center Wildland Fire Summary and Statistics Annual Report 2016,” National Interagency Coordination Center, 2016. Accessed: Apr. 20, 2021. [Online]. Available: https://www.predictiveservices.nifc.gov/intelligence/2018_statssumm/annual_report_2016.pdf

[13] National Interagency Coordination Center, “National Interagency Coordination Center Wildland Fire Summary and Statistics Annual Report 2017,” National Interagency Coordination Center, 2017. Accessed: Apr. 20, 2021. [Online]. Available: https://www.predictiveservices.nifc.gov/intelligence/2018_statssumm/annual_report_2017.pdf

[14] National Interagency Coordination Center, “National Interagency Coordination Center Wildland Fire Summary and Statistics Annual Report 2018,” National Interagency Coordination Center, 2018. Accessed: Apr. 20, 2021. [Online]. Available: https://www.predictiveservices.nifc.gov/intelligence/2018_statssumm/annual_report_2018.pdf

[15] A. Falk, A. Benda, P. Falk, S. Steffen, Z. Wallace, and T. B. Høeg, “COVID-19 Cases and Transmission in 17 K–12 Schools — Wood County, Wisconsin, August 31–November 29, 2020,” *MMWR Morb. Mortal. Wkly. Rep.*, vol. 70, no. 4, pp. 136–140, Jan. 2021, doi: 10.15585/mmwr.mm7004e3.
